# Addressing implementation considerations when developing universal interventions for speech, language and communication needs in the ordinary classroom: a protocol for a scoping review

**DOI:** 10.12688/hrbopenres.13249.1

**Published:** 2021-04-29

**Authors:** Aoife Gallagher, Carol-Anne Murphy, Johanna Fitzgerald, James Law

**Affiliations:** 1 Health Implementation Science and Technology Research Cluster, Health Research Institute, School of Allied Health, University of Limerick, Limerick, Ireland; 2 Department of Educational Psychology, Inclusive & Special Education, Mary Immaculate College, Limerick, Ireland; 3 School of Education, Communication and Language Sciences, Newcastle University, Newcastle upon Tyne, Newcastle upon Tyne, NE1 7RU, UK

**Keywords:** Implementation Science, inclusive education, disability, speech, language and communication needs

## Abstract

**Background:** Understanding the factors that influence the implementation of health interventions in the context of education is essential to improving outcomes for children and young people with speech and language needs (SLCN). Yet implementation considerations have not been adequately addressed when developing interventions for this context. The aim of this paper is to present a protocol for a scoping review of existing implementation frameworks that might guide SLCN intervention research in schools.

**Methods:** In accordance with scoping review guidelines, the study will be conducted in six phases: (1) identification of the research question, (2) identification of potentially relevant studies of Implementation Science frameworks, (3) study screening and selection, (4) charting and extracting data from identified frameworks, (5) collating, summarising and reporting the results and (6) consultation with stakeholders. Two reviewers will conduct the screening and extraction stages independently. Identified frameworks will be collated, and described, and constructs from the IS frameworks will be categorised using domains from the Consolidated Framework for Implementation Research. A draft IS model will be proposed based on the findings of the scoping review.

**Conclusions:** The findings of this review will provide guidance for researchers in addressing implementation considerations when developing universal interventions for SLCN in the ordinary classroom, and ultimately will contribute towards improving outcomes for this vulnerable childhood population.

## Introduction

According to school census data, children and young people with speech, language and communication needs (SLCN) represent a sizeable proportion of the school-aged population ( Lindsay & Strand, 2016; Norbury *et al.*, 2016a), and are a vulnerable group in terms of poor social and emotional, and educational outcomes ( Conti-Ramsden & Durkin, 2012; Conti-Ramsden *et al.*, 2018). Children and young people with SLCN can struggle to understand and/or to use grammar, to learn new vocabulary and linguistic concepts and/or to use language for higher order tasks such as making inferences and/or predictions and problem-solving ( Norbury *et al.*, 2016a) as well as co-occurring difficulties with written language ( Alloway *et al.*, 2017; Archibald *et al.*, 2013). As teaching and learning in the classroom is essentially language-based, children with SLCN can be particularly disadvantaged in accessing the curriculum ( Dockrell & Lindsay, 1998; Dockrell *et al.*, 2017). For some, these barriers to learning can have a negative impact into adulthood, with reported difficulties gaining skilled employment, mental health ( Botting *et al.*, 2016) and living independently ( Botting *et al.*, 2016). Prior to school entry, speech and language difficulties are considered a ‘health’ need and are managed by speech and language therapists (SLTs), employed by health services.

Once of school age, the majority of students with SLCN in Ireland, as in many other high-income countries, attend ordinary (mainstream) schools ( Cosgrove *et al.*, 2014; Gallagher *et al.*, 2020). ( Black-Hawkins *et al.*, 2016; Day & Prunty, 2015; Nilholm, 2020). Where a child’s SLCN act as a barrier to their learning and participation in the classroom, they are referred to as ‘special educational needs’ (SEN). In the Irish context, a special educational need is defined as ‘… a restriction in the capacity of the person to participate in and benefit from education on account of an enduring physical, sensory, mental health or learning disability, or any other condition which results in a person learning differently from a person without that condition’ (EPSEN Act 2004, p 6). The educational landscape for children and young people with SEN in Ireland has witnessed radical and rapid transformation since the early 1990s, in line with many countries internationally ( Department of Education and Skills, 2015), with a shift in focus towards creating more socially-responsive inclusive school environments ( Rose *et al.*, 2015). Essential to this policy goal is the effective integration of ‘health’ interventions in school ( IASLT, 2017; Rix *et al.*, 2013a).

For schools to address the needs of children and young people with SLCN, three distinct tiers of intervention exist; interventions delivered at a universal level (support for all); interventions delivered at a targeted level (support for some); and interventions delivered at a specialist level (support for few) ( Rix *et al.*, 2013b). Speech and language therapists provide interventions at all three levels. This tiered approach to the delivery of support in school is underpinned by public health principles ( Ehren & Nelson, 2005; Greenwood *et al.*, 2017) including the need for; early and accurate identification of needs, more equitable access to appropriate support ( Law *et al.*, 2013) and more efficient and cost-effective allocation of specialist resources ( Ebbels *et al.*, 2019; Lindsay *et al.*, 2012).

Considerable emphasis is placed on the provision of effective universal interventions in the classroom for children and young people with special educational needs in policy in Ireland ( Department of Education and Skills, 2017; National Council for Special Education, 2019). Universal interventions are defined as techniques or strategies that can be integrated into the teaching and learning of the classroom for the benefit of all students including the child with SEN ( Edyburn, 2010). In the field of SLT, intervention research has focused primarily on establishing the efficacy of therapy techniques for school-aged children with SLCN to be delivered at targeted and/or specialist levels ( Ebbels *et al.*, 2017; Walker *et al.*, 2020) with less focus on the development of interventions at a universal level ( Dobinson & Dockrell, 2021; Dockrell & Howell 2015; Ebbels *et al.*, 2017; Law *et al.*, 2012).

Universal level interventions to support SLCN which have been piloted in the classroom setting have paid limited, if any, attention to the contextual factors which may facilitate or hinder their implementation ( Douglas & Burshnic, 2019; Roberts *et al.*, 2020). Despite the fact that multiple and complex barriers to the uptake of evidence-based research into routine teaching practice have been reported for some time in the education literature ( Cook & Odom, 2013; Domitrovich *et al.*, 2008; Fixsen *et al.*, 2013), there is little to guide researchers in how to develop universal interventions for SLCN that are acceptable, sustainable, effective and implementable in the ordinary classroom.

According to Eccles & Mittman (2006), implementation science (IS) can facilitate the study of methods to support the systematic uptake of evidence into routine practice and by doing so can improve the quality and effectiveness of healthcare across contexts. The use of IS frameworks can guide the development of acceptable and sustainable interventions ( Bauer & Kirchner, 2020), and the evaluation of implementation processes, as well as to provide insights into the contextual factors that can support the adoption of research findings and/or to understand organisational and/or practice change at a theoretical level ( Bauer & Kirchner, 2020; Geng *et al.*, 2017). 

According to the findings of a recent systematic review of the IS literature ( Nilsen & Bernhardsson, 2019), there has been a large number of IS studies conducted in the last 20 years, most of which have been developed and tested within the context of healthcare settings. There have also been an increasing number of studies focussing on the implementation of school wide initiatives in the education literature particularly with regards to emotional behavioural needs ( Durlak & Wells, 1997; Durlak, 2016; Oberle *et al.*, 2016). In the field of SLT, researchers have outlined the potential benefits of IS in addressing research to practice gaps ( Campbell & Douglas, 2017; Douglas & Burshnic, 2019; Olswang & Prelock, 2015). However, there has been minimal focus on the use of IS frameworks when developing and implementing universal interventions for children with SLCN in the classroom ( Olswang & Prelock, 2015).

In this study we will scope the literature in order to map the use of IS frameworks in the development and/or implementation of *universal interventions* for children identified as having special educational needs in the ordinary classroom in general schools. The study findings will provide clarity and guidance for researchers in addressing implementation considerations when developing and implementing universal interventions for SLCN in this context. By developing more acceptable and sustainable universal interventions, we have the potential to improve the participation and achievement of children with SLCN in school.

The objectives of this review are to:

1. To map the use of IS frameworks in the implementation of universal interventions in the ordinary classroom for children and young people identified as having special educational needs in the published peer-reviewed literature
2. To describe the IS frameworks and constructs used including theoretical underpinnings where stated
3. To propose constructs that may be applicable to the development and/or the implementation of universal interventions for children and young people with SLCN

The research questions are:

- What implementation science frameworks have been used in the implementation of universal interventions in ordinary classrooms for children with special education needs?
- What specific constructs have been identified in the research literature as important when researching the development and implementation of universal interventions in this context?
- What constructs might be applied to research aimed at developing or implementing universal interventions for speech, language and communication needs in this context?

## Methods

A scoping review will be undertaken. Like systematic reviews, scoping reviews use a systematic approach to searching, screening, and reporting of the literature but differ in that the method is used to: examine the extent, range, and nature of a particular research activity, to summarize/map research findings, and/or to identify concepts which may be transferable to other research contexts. This method is therefore most suited to addressing the research questions of the study.

This review will be carried out in six phases as described by Levac *et al.* (2010). These phases include: (1) developing the research question, (2) identifying potentially relevant studies, (3) study screening and selection, (4) charting and extracting data from included papers, (5) collating, summarising and reporting the results and (6) consultation with stakeholders. As suggested by the researchers ( Levac *et al.*, 2010), more extensive content analysis may be required, depending on the nature of the papers included in the review.

### 1. Developing the research question

According to ( Colquhoun *et al.*, 2014) it is essential to have a well-defined research question which includes a clearly-defined phenomenon of interest, a well-defined population and a description of the context when conducting a scoping review given the potentially large body of papers which may be analysed. Our review aims to synthesise frameworks used in empirical studies which explicitly aim to address implementation considerations when developing and/or implementing universal level interventions for children and young people identified as having special educational needs in ordinary classrooms.

In this study, the *phenomenon of interest* is the use of *implementation science frameworks*. We define implementation science research as “the scientific study of methods to promote the systematic uptake of research findings and other evidence-based practices into routine practice” ( Eccles & Mittman, 2006, p1). We define a framework as per Nilsen’s definition i.e. “a structure, overview, outline, system or plan consisting of various descriptive categories, e.g. concepts, constructs or variables, and the relations between them that are presumed to account for a phenomenon” ( Nilsen, 2015, p2).

Given the focus of the research is *implementation* we avoid using narrow inclusion and exclusion criteria based on a particular diagnostic category when specifying our *population of interest*. Therefore, studies will include children with any of the following: (i) communication and interaction needs, (ii) cognition and learning needs, (iii) social, emotional and mental health difficulties needs, and (iv) sensory and/or physical needs. We will include intervention studies which aim to improve any of the areas of need stated above provided they are universal level and related to the general classroom.

As most CYPs with these needs are educated in general schools the *context of interest* in the study includes the general classroom. Given the differences in education systems internationally, we are aware that it will be important to pay careful attention to selecting search terms and to extracting as much detail as possible about the setting in which the study was conducted in order to contextualise our findings. 

### 2. Identifying potentially relevant studies

***Search strategy and terms.*** A search string will be developed based on an adapted Phenomenon - Situation (P-S) framework ( Jakubec & Astle, 2017). See Table 1 for the preliminary search string. The search results will be reported using the PRISMA extension for Scoping Reviews (PRISMA-ScR) tool ( Tricco *et al.*, 2018), the most up-to-date guidance on conducting scoping reviews. Search terms will be adapted to the basic search particulars (eg., wildcards (*) and truncations, capacity for complex searches) of each electronic database. Electronic searches will be supplemented with snowballing techniques such as by seeking recommended articles from well-cited IS researchers across health and education. Any articles retrieved via manual searches will be incorporated into the PRISMA-ScR flowchart. A manual search of reference lists will be undertaken as will a manual search of Implementation Science websites known to the researchers, for additional relevant papers.

**Table 1.** Search String Developed for CINAHL in EBSCO.

1	implementation N1 framework* OR implementation N1 model* OR “research utili?ation” N1 model* OR “research utili?ation” N1 framework* OR “knowledge translation” N1 framework* OR “knowledge translation” N1 model* OR “knowledge-to-action framework” OR “K2A framework” OR “KTA framework” OR “Quality Implementation Framework” OR “QIF” OR “promoting action on research implementation in health services” OR “PARIHS” OR “i-PARIHS” OR “active implementation framework” OR “AIF” OR “consolidated framework for implementation research” OR “CFIR” OR “theoretical domains framework” OR “TDF” OR “reach, effectiveness, adoption, implementation, and maintenance” OR “RE-AIM” OR “PRECEDE-PROCEED” OR “Understanding-User-Context Framework” OR “Stetler Model” OR “ACE star model of knowledge transformation” OR “Iowa model” OR “Ottawa Model” OR “Exploration Preparation Implementation Sustainment framework” OR “EPIS” OR “interactive systems framework” OR “integrated systems framework” OR ISF OR “Practical Robust Implementation and Sustainability Model” OR “PRISM” OR “stages of implementation framework” OR “stages of implementation completion” OR “implementation components model” OR “getting to outcomes” OR GTO OR “program implementation” OR (MH “Implementation Science”) OR (MH “Program Implementation”)
2	school* OR educati* OR class* OR “classroom-based” OR “school-based” OR (elementary OR grade OR middle OR primary OR secondary OR high) N3 (school* OR student* OR child* OR pupil*) OR kindergarten OR K-12 OR school-age OR “mainstreaming education” OR “mainstream* special education” OR “mainstreaming” OR “mainstream school” OR “regular class*” OR “mainstream class*” OR “mainstream education system” OR “inclusi* education” OR “integrati* education” OR (MH “Schools”) OR (MH “Education”) NOT “special school*” NOT “special class*” NOT “special unit*” NOT “language class*” NOT preschool*
3	“special need*” OR special OR “additional need*” OR “complex need*” OR disabilit* OR disabled OR impairment OR impaired OR “learning disabilit*” OR “learning disorder*” OR “LD” OR “learning impairment” OR “non-verbal learning disabilit*” OR “developmental academic disabilit*” OR “academic disorder*” OR “intellectual disabilit*” OR “ID” OR “developmental disease*” OR “developmental* disabilit*” OR “DD” OR “developmental disorder*” OR “child developmental disorders” OR “cognitive impairment*” OR “cogniti* disorder*” OR “mental retardation” OR “mentally retarded” OR “mental handicap” OR “mentally handicapped” OR “mental deficiency” OR “mental disorder” OR handicap* OR “communicati* disorder*” OR “communicate* impair*” OR “communicati* dysfunction*” OR “SLCN” OR “speech language communication need*” OR apraxia OR dyslexia OR dyspraxia OR “developmental language disorder* ” OR “DLD” OR “specific language impairment*” OR “SLI” OR “language development* disorder” OR “language disorder*” OR “language disabilit*” OR “emotional needs” OR “emotional disorder*” OR “social disabilit*” OR “autist*” OR ASD OR “autistic disorder” OR “autism spectrum disorder*” OR “pervasive developmental disorder” OR PDD OR “PDD-NOS” OR “reading disabilit*” OR “reading disorder*” OR “reading failure” OR “reading retardation” OR “retarded readers” OR dyslexia OR alexia OR “literacy need*” OR apraxia OR “developmental apraxia” OR “childhood apraxia of speech” OR CAS OR “childhood verbal apraxia” OR “developmental apraxia of speech” OR “developmental articulatory apraxia” OR “developmental verbal dyspraxia” OR DVD OR “dyspraxia of speech” OR “speech apraxia” OR “nonverbal learning disabilit*” OR “neurologic* disorder*” OR “congenital Impair*” OR “physical disabilit*” OR “motor dysfunction” OR “fine motor dysfunction” OR “cerebral palsy” OR “spina bifida” OR “down syndrome” OR “attention deficit disorder” OR ADD OR “attention deficit/hyperactivity disorder” OR ADHD OR “developmental coordination disorder*” OR DCD OR “developmental dyspraxia” OR “motor coordination disorder” OR “coordination disorder” OR “clumsy child*syndrome” OR “movement disorder” OR “motor skills disorder*” OR “muscular dystrop*” OR “congenital disorder*” OR “sensory system disorder*” OR “sensory disintegrative disorder” OR “sensory defensiveness” OR “sensory processing disorder” OR “anxiety” OR “global developmental delay” OR “cleft lip” OR “cleft palate” OR “cleft lip and palate” OR “orofacial cleft” OR (MH "Intellectual Disability") OR (MH "Developmental Disabilities") OR (MH "Dyslexia") OR (MH "Students, Disabled") OR (MH "Learning Disorders") OR (MH "Disabled") OR “at-risk”
4	1 AND 2 AND 3

Following preliminary assessment of electronic databases for their relevance and coverage of the topic literature, five electronic databases have been identified to be included in the search:

- ERIC
- EMBASE
- AMED
- PubMed
- PsycARTICLES

### 3. Study screening and selection

To ensure compatibility with the standards expected of a scoping review for peer-reviewed publication, explicit inclusion/exclusion criteria will be applied.

The following studies will be included:

- Empirical studies published in peer-reviewed journals
- Primary research with qualitative, mixed methods and/or quantitative design
- Related to universal interventions
- Related to interventions aimed at improving (i) communication and interaction needs, (ii) cognition and learning needs, (iii) social, emotional and mental health difficulties, and/or (iv) sensory and/or physical needs
- Related to the ordinary classroom setting
- Papers published/available in English

The following literature will be excluded:

- Policy briefs, books, book chapters, editorials, commentaries and published or unpublished reports from governments and other agencies
- Related to pre-school/kindergarten years (> 5 years) or third level education (<18 years+)
- Related to targeted/ specialist level interventions in general schools
- Related to special education settings/ special classrooms ^1^
- Not related to children and young people identified as having difficulties in one of the following: (i) communication and interaction, (ii) cognition and learning, (iii) social, emotional and mental health difficulties, and (iv) sensory and/or physical needs.

Once the searches are conducted, citation abstracts for all items will be exported into EndNote (x9). Rayyan (2016) -a web and mobile app for systematic reviews will be used to independently screen the papers. After removing duplicates, the remaining items will be screened for inclusion, initially on the basis of title and abstract. Where inclusion or exclusion cannot be determined on the basis of title and abstract, the paper will be included for full-text screening.

Two researchers (ALG and CAM) will screen an initial sample of randomly selected papers independently for inclusion. As suggested by ( Levac *et al.*, 2010), inclusion/exclusion criteria may be refined at this point if necessary. Any differences in screening decision-making will be discussed. If needed, independent screening will be continued until decisions are consistent. Full-text screening will then be undertaken between the researchers for all papers where it was not possible to determine inclusion or exclusions on the basis of title and abstract. Following the full-text screening, studies recommended for exclusion will be reviewed by an additional researcher (JF) to ensure consistency in the application of exclusion criteria. A final list of all included papers will be agreed amongst the research team.

### 4. Data extraction

Two researchers, (ALG and CAM) will simultaneously extract data into Excel (2019) for five randomly selected papers in order to assure consistency in data extraction (ALG and CAM). Following this check for quality assurance, which will be repeated until agreement is reached, we will divide the remaining included studies for data extraction. Data to be extracted will include:

- Background information related to the study (author(s), date, study objectives, research question, intervention details, country).
- Population(s)/ Special Educational Need of interest.
- Name and description of IS framework.
- Description/definitions of the constructs used in the study.
- Theoretical underpinnings of the research and/or framework if referenced.
- Any constructs identified as important to inform implementation research when developing universal interventions in ordinary classrooms.

As discussed by Levac *et al.* (2010), this process may need to be iterative meaning that new categories may need to be developed based on the findings of the extraction phase.

As the purpose of this scoping review is to map and synthesise the current research, papers will not be excluded based on quality criteria. However, part of the extraction process will include an assessment of methodological quality using the ‘Mixed Methods Appraisal Tool’ (MMAT) ( Pace *et al.*, 2012). This step has been included as the study does aim to make recommendations for future research. Any methodological weaknesses of studies will be discussed when presenting the findings and when making recommendations. 

### 5. Collating, summarising and reporting results

The analysis will be conducted by ALG and CAMin consultation with the review team. Descriptive statistics, if relevant, will be used to summarise the general characteristics of included studies. We will critically appraise all included studies using the validated Mixed Methods Appraisal Tool ( Pluye *et al.*, 2009), assigning a quality score to each paper and including this in the table. As we are mapping the current literature, we will not exclude on the basis of quality score. We will then conduct a qualitative content analysis to map the extracted IS constructs. Our analysis will be carried out deductively, guided by five IS domains developed by Damschroder *et al.* (2009). These domains have been widely used in implementation science research across a range of health service research contexts previously. The domains relate to *intervention characteristics, inner setting*, *outer setting*, *and implementation process*. These domains will be operationalised as per definitions set out by Damschroder *et al.* (2009); *intervention characteristics* will include any constructs related to the features of the intervention that are stated to have influenced implementation, *inner setting* will include organisational factors which have been reported to influence implementation, *outer setting* will include elements related to the broader context or environment that are stated to have influenced implementation such as policy, *characteristics of individuals* involved in the implementation which impacted the success of the implementation and the implementation process includes any identified strategies or tactics reported to have influenced implementation.

### 6. Expert consultation

A summary of study aims and findings will be shared with a sample of IS researchers and practitioners who support speech and language needs in school (teachers, educational psychologists and speech and language therapists who work in schools. Researchers will be identified via known research networks and by reviewing relevant IS journals. Practitioners will be identified via professional networks. Feedback from these stakeholders will be used to shape the final interpretation and presentation of the study findings. Findings of the scoping review will be disseminated by publication and at relevant conferences internationally.

## Discussion

Children and young people with speech, language and communication needs (SLCN) represent a sizeable proportion of the school-aged population ( Lindsay & Strand, 2016; Norbury *et al.*, 2016b). Such needs can have a negative impact on an individual’s educational outcomes and potentially longer term in terms of employability and difficulties with mental health ( Conti-Ramsden & Durkin, 2012; Conti-Ramsden *et al.*, 2018).

Interventions for children and young people with SLCN in school are delivered using a phased approach; at a universal level, then a targeted level and a specialist level depending on the child’s response to intervention. Intervention research in SLCN has focused mainly on establishing the efficacy of interventions aimed at a targeted or specialist level, with insufficient consideration given to the development and/or implementation of interventions to support SLCN, delivered at a universal level, in the ordinary classroom. Understanding the factors which can facilitate and/or act as a barrier to the implementation of universal interventions is essential if we are to improve outcomes for this population.

We will conduct a scoping review of the implementations science (IS) literature to map and synthesise the use of IS frameworks in developing and/or implementing universal interventions in school. By synthesising the use of existing IS frameworks and proposing an IS model, we will provide clarity and guidance for researchers in addressing implementation considerations when developing interventions for SLCN in the classroom. Doing so has the potential to support the uptake of interventions for SLCN in school where efficacy has already been established, as well as to guide acceptable and sustainable intervention development in this context going forward.

### Strengths and limitations of the proposed study

This scoping review protocol is the first to focus on implementation considerations in the development of universal interventions for SLCN in the ordinary classroom. We will use the Preferred Reporting Items for Systematic Reviews and Meta-Analyses extension for Scoping Reviews (PRISMA-ScR) tool, and the most current guidance on conducting scoping reviews, in order to ensure a systematic approach to searching, screening and reporting. This study will search journals from across the fields of education and health in order to maximise the comprehensiveness of the review. This scoping review may miss studies published outside of journals (e.g. theses, book chapters, unpublished reports and other grey literature).

### Study status

The search strategy is being finalised currently for this scoping review.

## Data availability

No data are associated with this article.
