# Supplementary material for: Addressing implementation considerations when developing universal interventions for speech, language and communication needs in the ordinary classroom: a protocol for a scoping review
Source: HRB Open Res. 2022 Jan 31;4:41. Originally published 2021 Apr 29. [Version 3] doi: 10.12688/hrbopenres.13249.3 (PMC8812329; doi:10.12688/hrbopenres.13249.3)
Supplement: Peer review history

# Peer review: Reviewer response for version 3

**DOI:** 10.21956/hrbopenres.14692.r31369

**Published:** 2022-02-02

**Authors:** Trina D Spencer

**Affiliations:** 1 Department of Child and Family Studies, University of South Florida, Tampa, Florida, USA

I approve this article, however, I have some very small edits that should be attended to.

 The abstract is much clearer and accurately reflects the Method featured in the review. There is an extra space after the 2) screening...

 First paragraph of intro: "as well as co-occurring difficulties with written language." should be, "as well as **experience **co-occurring difficulties with written language." Grammatically consistent with the subject of the sentence.

 The intro is much clearer and the critical terms have been defined. Nice work.

 "...such as practitioners views..." the word practitioners needs an apostrophe to show possession: "practitioner **s'** views"

 Thank you for covering CFIR and why you believe this review is necessary. I also like the purpose of a scoping review and how it aligns with the current research questions. The intro is very well written.

 I believe the heading Methods should be Method. The phases are nicely described and it is clear what has already been completed and what you will do during the review.

 This is very long and excessively complex sentence. Please consider revising it. "In developing our research questions we were guided by the need to have a well-defined research question which includes a clearly-defined phenomenon of interest, a well-defined population and a description of the context when conducting a scoping review given the potentially large body of papers which may be analysed ( Colquhoun *et al.*, 2014).

 I suggest rewriting this sentence, "...screening will be continued until decisions are consistent." to be "...screening will continue until decisions are consistent."

 The precision of the phases is very well done. The process the authors will engage in to complete this review are very clear and understandable. I enjoyed reading this version very much. I appreciate the specification and where there will likely be refinement. Thank you for your efforts to improve this protocol. It will make a great contribution and I look forward to your findings, as it will help me in my work too.

 I express my condolences for the passing of James Law. That is a great loss for you as his colleagues and for the field. My deepest sympathies. May you always remember the lessons he taught you.

Is the study design appropriate for the research question?

Partly

Is the rationale for, and objectives of, the study clearly described?

Partly

Are sufficient details of the methods provided to allow replication by others?

Partly

Are the datasets clearly presented in a useable and accessible format?

Not applicable

Reviewer Expertise:

Multi-tiered oral language interventions; implementation research; evidence-based practice.

I confirm that I have read this submission and believe that I have an appropriate level of expertise to confirm that it is of an acceptable scientific standard.

# Peer review: Reviewer response for version 2

**DOI:** 10.21956/hrbopenres.14667.r30844

**Published:** 2021-11-26

**Authors:** Michael Donnelly

**Affiliations:** 1 Centre for Public Health, Queen's University Belfast, Belfast, UK

The revised version reads well to me. Good luck with your review.

Is the study design appropriate for the research question?

Yes

Is the rationale for, and objectives of, the study clearly described?

Yes

Are sufficient details of the methods provided to allow replication by others?

Yes

Are the datasets clearly presented in a useable and accessible format?

Yes

Reviewer Expertise:

Public health and health care research

I confirm that I have read this submission and believe that I have an appropriate level of expertise to confirm that it is of an acceptable scientific standard.

# Peer review: Reviewer response for version 2

**DOI:** 10.21956/hrbopenres.14667.r30843

**Published:** 2021-11-16

**Authors:** Trina D Spencer

**Affiliations:** 1 Department of Child and Family Studies, University of South Florida, Tampa, Florida, USA

Although the authors have reconciled many of the previous problems, there are additional problems that should be taken care of. In particular, the use of acronyms/abbreviations is horribly inconsistent. Please choose one way to do it and keep them consistent throughout. The second is how to understand the first phase of the scoping review when the report includes research questions. See comments below.

 ABSTRACT

 It is not clear what the letters SLCN abbreviate.

 (1) identification of the research question...does this refer to the research questions addressed in the various studies reviewed or the research question of the proposed scoping review?

 In the Methods of the Abstract, the phases are not consistent. For example, (3) what are you doing with the study screening and selection? There is no verb in this phase.

 "stages" or "phases" These should be consistent throughout.

 INTRODUCTION

 Here the abbreviation SLCN is specified correctly.

 I think you need : instead of ; in the following sentence "including the need for; early and accurate identification..."

 There is inconsistent use of acronyms, capitalization, and the full words (e.g., Implementation Science vs. IS and implementation science).

 2. Objective has the word "including" twice. Please rewrite the sentence to avoid that.

 It is still not clear what "constructs" the authors are thinking about. The intro doesn't offer more information. It is very vague.

 METHODS

 The last line of the first paragraph uses "This" but the previous sentence is talking about differences. What does "this" refer to?

 Just as in the Abstract, the six phases are not listed well. (1) Developing the research question - What are we talking about? You have three research questions in your introduction so why would the first phase be for you to develop your research question? What is going to happen in phase (3)? There is no verb, unless "study" is the verb and it is very vague.

 If you are going to use the acronyms, use them throughout.

 They are sometimes abbreviated SLCN and sometimes written out. What are CYPs? The authors' use of acronyms are so inconsistent.

 The mismatch between what the Method says and what the authors have already done (e.g., established research questions) is very difficult to reconcile.

 There are so many acronym problems.

Is the study design appropriate for the research question?

Partly

Is the rationale for, and objectives of, the study clearly described?

Partly

Are sufficient details of the methods provided to allow replication by others?

Partly

Are the datasets clearly presented in a useable and accessible format?

Not applicable

Reviewer Expertise:

Multi-tiered oral language interventions; implementation research; evidence-based practice.

I confirm that I have read this submission and believe that I have an appropriate level of expertise to confirm that it is of an acceptable scientific standard, however I have significant reservations, as outlined above.

# Response

**Published:** 2021-12-04

**Authors:** Aoife Gallagher

**Affiliations:** University of Limerick, Ireland

In V3, we have amended the document following on from some further comments from Reviewer  2.

 Our responses to each comment are set out below.

 Specifically:

 ABSTRACT

 (1) It is not clear what the letters SLCN abbreviate.

 Amended.

 (2) identification of the research question...does this refer to the research questions addressed in the various studies reviewed or the research question of the proposed scoping review?

 The word ‘proposed’ has been added for clarity. The five remaining phases to be completed are now described.

 (3) In the Methods of the Abstract, the phases are not consistent. For example, (3) what are you doing with the study screening and selection? There is no verb in this phase.

 Amended.

 "stages" or "phases" These should be consistent throughout.

 Now consistently referred to as Phases.

 INTRODUCTION

 (4) I think you need : instead of ; in the following sentence "including the need for; early and accurate identification..."

 Amended.

 (5) There is inconsistent use of acronyms, capitalization, and the full words (e.g., Implementation Science vs. IS and implementation science).

 We have reduced the use of acronyms to SLCN (speech, language and communication needs) and SEN (special educational needs) only. We use implementation science throughout in the place of IS.

 (6) Objective has the word "including" twice. Please rewrite the sentence to avoid that.

 Amended.

 (7) It is still not clear what "constructs" the authors are thinking about. The intro doesn't offer more information. It is very vague.

 We had detailed that we are interested to map determinant frameworks and their associated constructs in the methods section. Second paragraph, last sentence:

*“As we are scoping the use of determinant frameworks, the constructs we will report on are descriptive categories or factors believed or found to influence implementation outcomes (* *Nilsen, 2015* *)”*

  We have added this same sentence to the introduction.

 (8) METHODS

 The last line of the first paragraph uses "This" but the previous sentence is talking about differences. What does "this" refer to?

 Amended.

 (9) Just as in the Abstract, the six phases are not listed well. (1) Developing the research question - What are we talking about? You have three research questions in your introduction so why would the first phase be for you to develop your research question? What is going to happen in phase (3)? There is no verb, unless "study" is the verb and it is very vague.

 In order for the protocol to be publishable RQs and a sample search string needed to be developed. We have described the first phase as completed and the remaining phases to be completed.  

 (10) If you are going to use the acronyms, use them throughout. They are sometimes abbreviated SLCN and sometimes written out. What are CYPs? The authors' use of acronyms are so inconsistent.

 Addressed see point 5.

 (11) The mismatch between what the Method says and what the authors have already done (e.g., established research questions) is very difficult to reconcile.

 Addressed see point 9.

 (12) There are so many acronym problems.

 Addressed  see points 5.

 Thank you for taking the time to review this version of our paper.

# Peer review: Reviewer response for version 1

**DOI:** 10.21956/hrbopenres.14412.r30043

**Published:** 2021-09-14

**Authors:** Trina D Spencer

**Affiliations:** 1 Department of Child and Family Studies, University of South Florida, Tampa, Florida, USA

This is a study protocol for a scoping review on implementation science frameworks as they are applied to studies of speech, language, and communication interventions in mainstream classrooms. Generally, I am in favor of this protocol, but there are a number of unsettling aspects. I offer the following recommendations for the authors to consider.

1. The introduction needs a stronger rationale for why the researchers are interested in IS frameworks. The definition and utility of frameworks should be in the intro. It is not clear why they are choosing to look at frameworks as opposed to implementation studies generally. What if specific researchers do not specify a framework? And really, is it the framework that is important or is the research that is conducted? What does it mean and what does it look like to "apply a framework"? How is this different or better than just conducting the study? Likewise, the term "constructs" is so general and unspecified, yet they are critical in the research questions. Specification of the CFIR domains can help if they are introduced earlier. In other words, state you are using a specific framework in your study and give a rationale for its selection.
2. I am concerned about the introduction and discussion focusing on children with SLCNs but then the actual method is to include all children with disabilities. Alignment is needed and this is a pretty big thing. You can't do one thing and draw conclusions about another thing.
3. I recommend avoiding informal language (e.g., "map" as a verb) and other non-specific and non-descriptive language. 
4. It appears in the exclusion criteria that they are excluding any study with children ages 5-18 years old. Please review that content for accuracy.
5. CFIR is mentioned specifically in the abstract, but then only referenced without naming it in the protocol.
6. As a reader, I am not convinced that knowing what frameworks other researchers chose (if they stated it) is going to help future researchers. Be explicit about this.
7. Please read and edit carefully.

Is the study design appropriate for the research question?

Partly

Is the rationale for, and objectives of, the study clearly described?

Partly

Are sufficient details of the methods provided to allow replication by others?

Partly

Are the datasets clearly presented in a useable and accessible format?

Not applicable

Reviewer Expertise:

Multi-tiered oral language interventions; implementation research; evidence-based practice.

I confirm that I have read this submission and believe that I have an appropriate level of expertise to state that I do not consider it to be of an acceptable scientific standard, for reasons outlined above.

# Response

**Published:** 2021-10-28

**Authors:** Aoife Gallagher

**Affiliations:** University of Limerick, Ireland

Response to Reviewer 2

 Thank you so much for taking the time to provide us with constructive comments to improve our study. 

 Our responses to your most helpful comments are written under each, in italics.

 a. The introduction needs a stronger rationale for why the researchers are interested in IS frameworks.

*Now included.*

 b. The definition and utility of frameworks should be in the intro. It is not clear why they are choosing to look at frameworks as opposed to implementation studies generally. What if specific researchers do not specify a framework?

*Amended.*

 c. What does it mean and what does it look like to "apply a framework"? How is this different or better than just conducting the study? Likewise, the term "constructs" is so general and unspecified, yet they are critical in the research questions.

*Definitions have been added in relation to both points.*

 d. Specification of the CFIR domains can help if they are introduced earlier. In other words, state you are using a specific framework in your study and give a rationale for its selection.

*Now included in the protocol introduction*

 e. I am concerned about the introduction and discussion focusing on children with SLCNs but then the actual method is to include all children with disabilities. Alignment is needed and this is a pretty big thing. You can't do one thing and draw conclusions about another thing.

*We have added a justification for this in the methods and discussion. The premise of the study is based on the idea that we can learn from other Implementation Science work in schools as a starting point for considering such issues when working with children with SLCN.*

 f. I recommend avoiding informal language (e.g., "map" as a verb) and other non-specific and non-descriptive language. 

*We have changed some of the language accordingly.*

 g. It appears in the exclusion criteria that they are excluding any study with children ages 5-18 years old. Please review that content for accuracy.

*Corrected.*

 h. CFIR is mentioned specifically in the abstract, but then only referenced without naming it in the protocol.

 Corrected.

 i. As a reader, I am not convinced that knowing what frameworks other researchers chose (if they stated it) is going to help future researchers. Be explicit about this.

*We have justified this further in the introduction.*

 j. Please read and edit carefully.

*Done.*

 We are grateful for your time and thank you for your comments. We believe these have improved the reporting quality of the study protocol.

# Peer review: Reviewer response for version 1

**DOI:** 10.21956/hrbopenres.14412.r29879

**Published:** 2021-09-06

**Authors:** Michael Donnelly

**Affiliations:** 1 Centre for Public Health, Queen's University Belfast, Belfast, UK

The authors are proposing appropriately to use the well tried and tested Arskey and O'Malley scoping review methodology to collate studies that have used implementation frameworks in order guide the implementation of SLCN interventions in schools. The reporting of the review will follow the PRISMA guidance for scoping reviews. A further positive feature is the inclusion of stage 6 in the scoping review framework (omitted in many published scoping reviews). 

 There are references in the protocol to the development of interventions - there is need to clarify how intervention frameworks might be used to develop interventions (as well as guide the implementation in practice of existing evidence-based interventions).

 Will the review exclude studies that investigate implementation of interventions but does not include an implementation science framework? 

 Most readers will be familiar with EMBASE and PUBMED. Consider adding a sentence about the content of each database and the rationale for its selection. Is PSYCHINFO included in PsychArticles? Are social science databases included?

 How will websites be identified and how will data from websites be included in the review?

 Explain how the results of the appraisal of the methodological quality of each study will be used in the analysis and synthesis of the implementation frameworks particularly studies that the checklist deem to be of low quality. 

 Has consideration been given to the use in the conduct of the scoping review to one or more of the checklists that focus on implementation studies? 

 Hope that these reflections are helpful.

Is the study design appropriate for the research question?

Yes

Is the rationale for, and objectives of, the study clearly described?

Yes

Are sufficient details of the methods provided to allow replication by others?

Yes

Are the datasets clearly presented in a useable and accessible format?

Yes

Reviewer Expertise:

Public health and health care research

I confirm that I have read this submission and believe that I have an appropriate level of expertise to confirm that it is of an acceptable scientific standard, however I have significant reservations, as outlined above.

# Response

**Published:** 2021-10-28

**Authors:** Aoife Gallagher

**Affiliations:** University of Limerick, Ireland

Response to Reviewer 1

 Thank you so much for taking the time to provide us with constructive comments to improve the quality of our study. 

 Re 1. There are references in the protocol to the development of interventions - there is need to clarify how intervention frameworks might be used to develop interventions (as well as guide the implementation in practice of existing evidence-based interventions).

 Response: We have added text in the introduction to clarify how intervention frameworks might be used to develop interventions

 Re 2. Will the review exclude studies that investigate implementation of interventions but does not include an implementation science framework? 

 Response: Yes we will exclude studies without explicit reference to an IS framework. This has been clarified and rationale is given in the amended text.

 Re 3. Most readers will be familiar with EMBASE and PUBMED. Consider adding a sentence about the content of each database and the rationale for its selection.

 Response: These sentences have been added and tracked.

 Re 4. Is PSYCHINFO included in PsychArticles?

 That is our understanding, yes.

   

 Re 5. Are social science databases included?

 Yes – see tracked sentences related to the database descriptions.

 Re 6. How will websites be identified and how will data from websites be included in the review?

 Response: Further text has been added to clarify this point.

 Re 7. Explain how the results of the appraisal of the methodological quality of each study will be used in the analysis and synthesis of the implementation frameworks particularly studies that the checklist deem to be of low quality. 

 Response: We have added text to make clear how we will trace the quality of the studies in the analysis and synthesis. 

 Re 8.Has consideration been given to the use in the conduct of the scoping review to one or more of the checklists that focus on implementation studies? 

 Response: We were not completely sure whether the checklist referred to here relates to the appraisal of included studies. Having looked at the ‘Standards for Reporting Implementation Studies (StaRI) Statement’ (Pinnock et al 2017), however, we can see the benefit of including an appraisal of the reporting quality of the IS papers included in the analysis in addition to the methodological quality (planned to be assessed by the MMAT). We have therefore included reference to this checklist in the protocol. We would welcome further guidance from the reviewer on this point as needed.
